# Mechanisms of Vein of Marshall-Related Tachyarrhythmias and the Impact of Ethanol Infusion

**DOI:** 10.31083/j.rcm2504112

**Published:** 2024-03-25

**Authors:** Masateru Takigawa, Claire Martin, Pierre Jaïs

**Affiliations:** ^1^Department of Cardiac Pacing and Electrophysiology, Bordeaux University Hospital (CHU), 33000 Bordeaux, France; ^2^IHU Liryc, Electrophysiology and Heart Modelling Institute, Univ. Bordeaux, 33000 Bordeaux, France; ^3^Department of Cardiovascular Medicine, Tokyo Medical and Dental University, 113-8510 Tokyo, Japan; ^4^Department of Advanced Arrhythmia Research, Tokyo Medical and Dental University, 113-8510 Tokyo, Japan; ^5^Department of Cardiology, Royal Papworth Hospital, Papworth Road, CB2 0AY Cambridge, UK; ^6^Department of Medicine, Cambridge University, CB2 1TN Cambridge, UK

**Keywords:** ligament of Marshall, vein of Marshall, ethanol infusion, chemical ablation

## Abstract

The Ligament of Marshall (LOM) is a remnant of the embryonic sinus venosus and the left cardinal vein, containing a combination of fat, fibrous tissue, blood vessels, muscle bundles, nerve fibers, and ganglia. Various muscular connections exist between the LOM and the left atrium (LA) and the coronary sinus (CS). The LOM is richly innervated by autonomic nerves, with ganglion cells distributed around it. The unique characteristics of the LOM are responsible for generating focal electrical activities and enable it to serve as a substrate for micro- and macro-reentrant circuits. This, in turn, leads to the initiation and perpetuation of atrial fibrillation (AF) and atrial tachycardia (AT). Endocardial ablation in this region does not consistently succeed due to anatomical constraints within the left lateral LA, including the presence of a thicker and longer mitral isthmus (MI), anatomical variations between the MI and epicardial structures such as the CS and vein of Marshall (VOM) and circumflex artery, and the presence of fibrofatty tissue insulating the LOM. Furthermore, epicardial ablation is challenging for inexperienced institutions because of its invasive nature. Ethanol infusion into the VOM (EI-VOM) represents an effective and safe approach that can be employed in conjunction with radiofrequency ablation to eliminate this arrhythmogenic structure.

## 1. Introduction

**Fig. 1.**
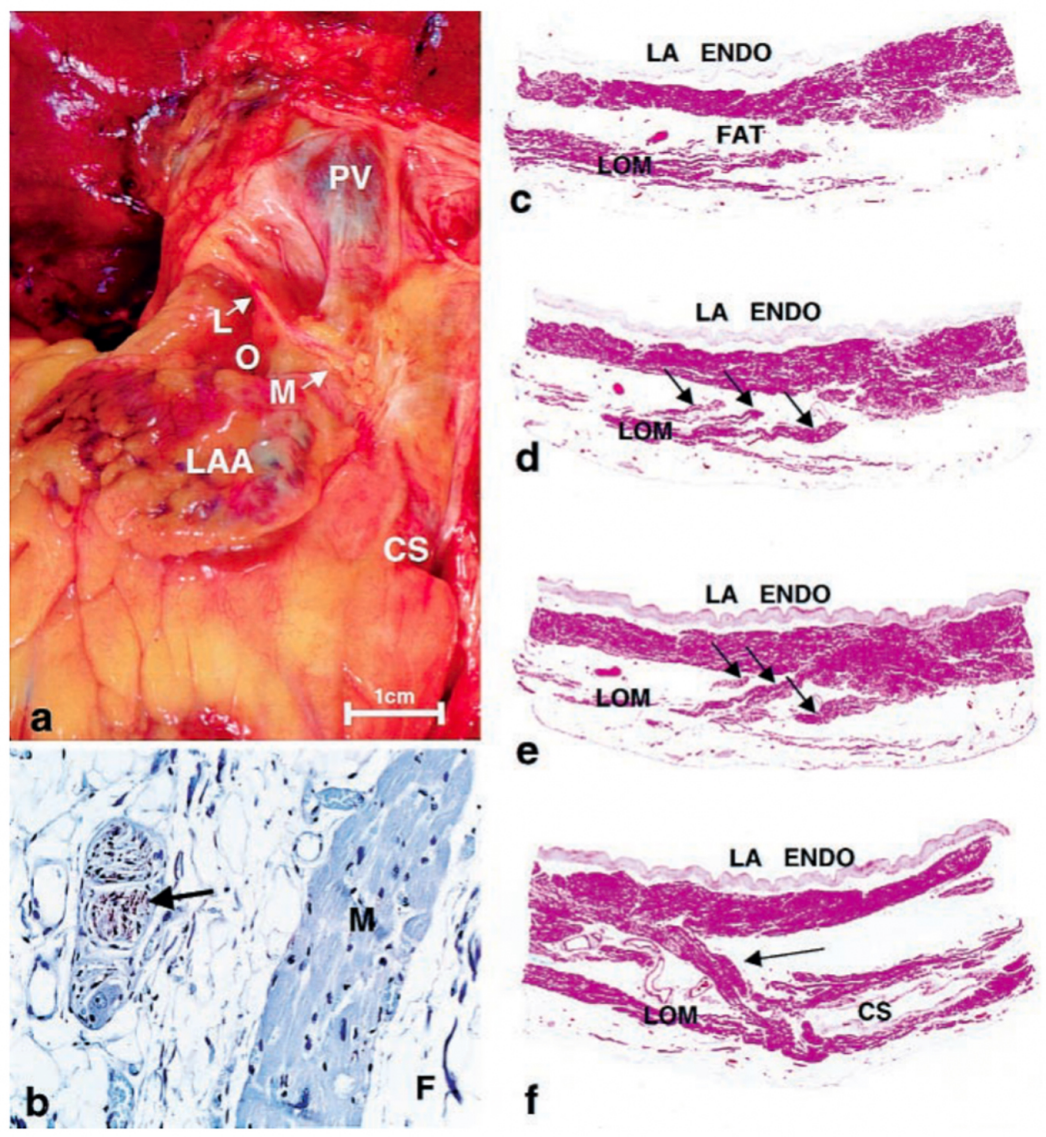
**Histology of Ligament of Marshall**. (a) Gross photo showing location on posterior surface of heart. (b) Immunohistochemical staining for tyrosine hydroxylase showing positively in nerve (brown staining—arrow). M, myocardium; F, fat. (avidin-biotin-peroxidase, ×120). (c–e) Subserial sections showing the LOM, isolated from the left atrial wall (in c), with 3 tracts (arrows) emerging from it (in d) and eventually inserting into the atrial wall (in e) (hematoxylin and eosin [H&E] stain ×10). (f) Section from lower end of LOM showing tract inserting into the left atrial wall (arrow) and coronary sinus (CS) (H&E stain ×10). This figure is reproduced from the original manuscript [3] with permission from Elsevier. CS, coronary sinus; LAA, left atrial appendage; LOM, ligament of Marshall; PV, pulmonary vein; LA ENDO, left atrial endocardium.

In 1850, John Marshall [1] was the first to describe a ligamentous structure in the left atrium (LA) located between the inferior and superior left pulmonary veins (PVs). This structure comprises multiple pericardial layers, fibrous tissue, fat, blood vessels and nerves, and is now called as the Ligament of Marshall (LOM). A comprehensive electrophysiological examination was conducted by Scherlag BJ *et al*. [2] in 1972. This examination demonstrated that the LOM serves as a terminal, insulated tract activated through an interatrial pathway that connects the posterior-inferior region of the right atrium to the posterior-inferior LA, running along the coronary sinus (CS). In this early investigation, it was observed that the terminal end of the LOM did not appear to reestablish connections with atrial musculature. Subsequently, in 2000, a comprehensive examination of the gross anatomical and microscopic characteristics of the LOM was conducted in seven postmortem human hearts by Kim DT *et al*. [3]. Their findings revealed that the human LOM: (1) receives innervation from sympathetic nerve fibers; (2) exhibits greater complexity compared to the canine LOM, characterized by the presence of ganglia, multiple sympathetic nerve fibers, numerous myocardial bundles, and blood vessels; (3) features multiple myocardial tract insertions into the CS and LA free-wall, all insulated by fibro-fatty tissue. These observations suggest the potential for the LOM to serve as a substrate for arrhythmias (Fig. 1, Ref. [3]).

In recent years, the importance of the LOM’s role in atrial fibrillation (AF) [4, 5, 6, 7, 8, 9, 10], and in scar-related atrial tachycardia (AT) [11, 12, 13] has garnered increasing attention. While LOM ablation may offer an effective and complementary therapeutic option for these arrhythmias, achieving transmural ablation of this epicardial structure from the endocardial side alone can be challenging. Ethanol-infusion to the vein of Marshall (EI-VOM) is a complementary and promising strategy to produce a durable lesion in this area. In this review, we investigate the anatomy and electrophysiological features of the LOM, its involvement in atrial arrhythmias, and strategies for eliminating this arrhythmogenic substrate through radiofrequency ablation and EI-VOM.

### Anatomy of the LOM — Musculature, Veins and Neurons

**Fig. 2.**
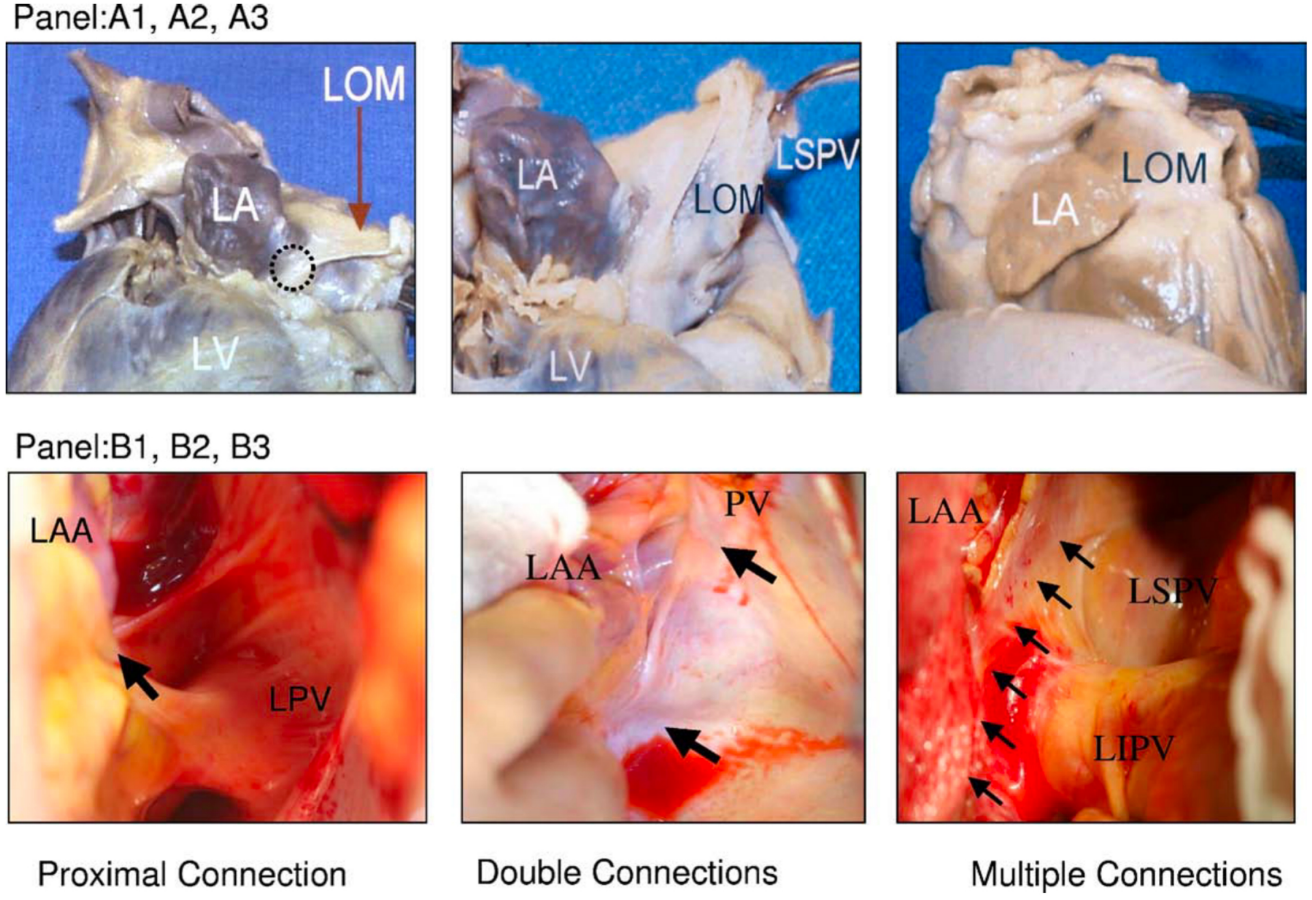
**Variations of LOM anatomy**. (A1–A3) Pictures from autopsy specimens. (B1–B3) Pictures taken during surgery. A black circle in (A1) indicates the proximal connection of the LOM to the CS. (A2) A different view of the same heart. The distal end of the LOM inserts into the LSPV. (A3) A second heart in which LOM was completely attached to the epicardium. A discrete ligament was not identified. (B1) Proximal connection (arrow) between the LOM and the CS. (B2) Both the proximal and distal connections of the LOM (2 arrows) in a second heart. (B3) A third heart, which seems to have multiple muscle fibers (arrows) connecting the LOM and the LA. CS, coronary sinus; LA, left atrium; LAA, left atrial appendage; LIPV, left inferior pulmonary veins; LOM, ligament of Marshall; LSPV, left superior pulmonary vein; LV, left ventricle; PV, pulmonary vein; LPV, left pulmonary vein. This figure is reproduced from the original manuscript [15] with permission from Elsevier.

The LOM is composed of residual tissues originating from the embryonic sinus venosus and the left cardinal vein, encompassing adipose tissue, fibrous structures, muscle bundles, blood vessels, ganglia, and nerve fibers [1, 3, 14]. Diverse muscular connections are observed between the LOM and the LA or CS [14, 15] (Fig. 2, Ref. [15]), but typically, the LOM can be anatomically subdivided into proximal, mid, and distal portions [15]. The proximal segment establishes a direct connection with the muscle sleeve of the CS. The middle segment of the LOM links to the left lateral ridge and the left PVs. Extending beyond the left PVs, the distal segment, in certain instances, inserts into the free wall of the LA. Makino M *et al*. [14] reported the observation of continuously extending multiple and broad connections between the LA and the LOM in approximately one-third of cases.

The vein of Marshall (VOM), one of the largest veins in the LA, is insulated within the LOM. It drains the posterior and posterolateral walls of the LA, coursing obliquely and inferiorly towards the CS, with its ostium situated within the proximal CS, just proximal to the Vieussens valve. This vein may be identifiable in more than 90% of cases through CS venography [16].

Neural composition is another vital component of the LOM. Immuno-histochemical examinations of the LOM indicate rich innervation by autonomic nerves and ganglia. In a study conducted by Doshi R *et al*. [9] in 1999, involving 12 canine atria, it was demonstrated that the LOM comprises muscle tracts and abundant nerve bundles primarily consisting of well-insulated sympathetic nerves within fibrofatty tissues. Conversely, Ulphani J *et al*. [17] in 2007 documented in a study involving 10 dogs that the LOM predominantly comprises parasympathetic nerve fibers, which originate from the left vagus, traverse through the LOM, and provide innervation to several structures in the posterior LA. These conflicting findings were explained by Makino M *et al*. [14] in postmortem human hearts. They showed that sympathetic nerve fibers were densely concentrated around the PV-LA junctions, with parasympathetic ganglia primarily distributed at the CS juncture. From the distal to the proximal segments of the LOM, the sympathetic nerve fibers gradually decrease while the parasympathetic ganglions increase.

Yu X *et al*. [18] conducted a study in 16 dogs, employing 6-hour rapid atrial pacing (20 Hz, 2 × threshold) both before and after selectively ablating the distal section of the LOM to examine its impact on atrial electrical remodeling. The ablation of the distal LOM led to a decrease in sympathetic indices of heart-rate variability and serum norepinephrine levels. Post-pacing, there were observed alterations such as shortened atrial effective refractory period (ERP), increased ERP dispersion, and heightened inducibility and persistence of AF. However, subsequent ablation of the distal LOM reversed these effects. Conversely, preemptive ablation of the distal LOM before rapid atrial pacing inhibited changes in ERP and prevented the induction and sustenance of AF. These findings suggest that the therapeutic and defensive effects of distal LOM ablation could be linked to the reduction of cardiac sympathetic activity, thereby exerting global effects on atrial tissue [18].

**Fig. 3.**
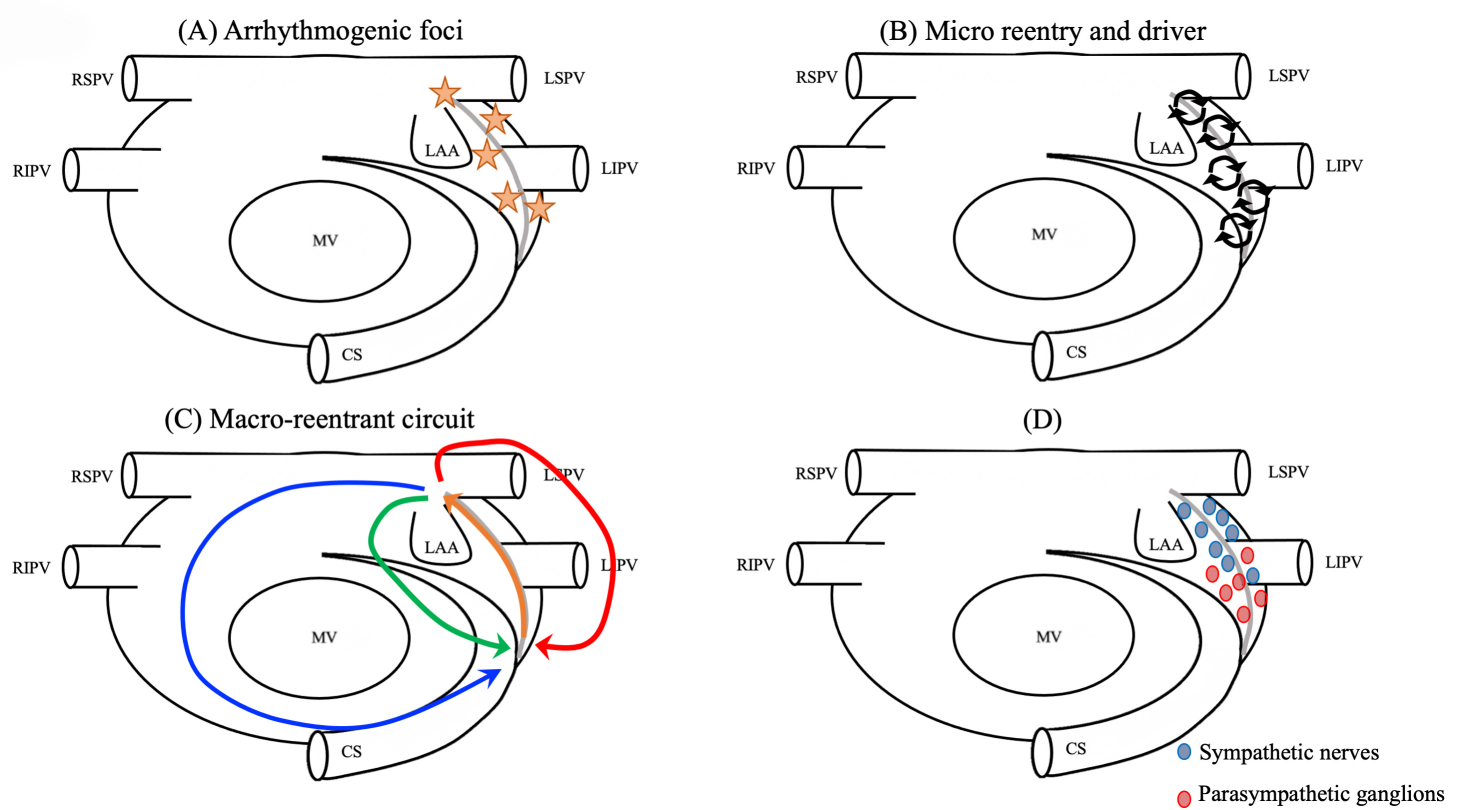
**Mechanism of LOM-related tachyarrhythmias**. (A) The source of triggers. (B) Drivers or micro-reentries forming the substrates of reentry. (C) Macroreentrant or localized circuits totally or partially using the LOM. (D) Rich autonomic innervation (e.g., parasympathetic ganglions around the ostium of the VOM and adrenergic nerves around the distal sympathetic nerves). CS, coronary sinus; LAA, left atrial appendage; LIPV, left inferior pulmonary vein; LSPV, left superior pulmonary vein; MV, mitral valve; RIPV, right inferior pulmonary vein; RSPV, right superior pulmonary vein; LOM, ligament of Marshall; VOM, vein of Marshall.

Not only the histological and experimental data mentioned above, but also the clinical findings reported explain the relation between the arrhythmogenicy of LOM and the mechanism of AF/AT initiation and perpetuation of these arrhythmias [4, 5, 6, 7, 8, 9, 10, 11, 12, 13, 14, 15, 16, 17, 18] (Fig. 3).

## 2. Role of the LOM as a Focus for AF

The LOM as an arrhythmogenic source was first reported in an experimental study by Scherlag BJ *et al*. [19]. Multiple subsequent case reports and studies have shown ectopic beats originating from the LOM as triggers for paroxysmal AF [4, 20, 21, 22]. Stimulation of left cardiac sympathetic nerve in dogs has been reported to induce an ectopic atrial rhythm originating from the LOM area. Additionally, the LOM has been observed as a potential source of focal atrial tachycardia (AT) [23] and AF [4, 5, 6]. Hwang C *et al*. [4] successfully documented double potentials in the VOM. The origin of AF was observed in the muscle bundle within the LOM in six patients in whom VOM electrograms were directly recorded. Ablation targeting the insertion site of the VOM effectively terminated AF in four of these patients. These findings suggest that the LOM may serve as the origin of focal AF in certain individuals. Katritsis D *et al*. [5], demonstrated the feasibility of recording Marshall potentials and a significant reduction in the frequency of adrenergic AF burdens and symptoms by the abolition of these activities. Depending on the types of connections between the LOM and LA or CS, electrograms of the VOM are sometimes discretely visible, as clearly demonstrated by Han S *et al*. [20], and sometimes not. Lin WS *et al*. [24], studied 240 patients with a total of 358 ectopic foci initiating Paroxysmal AF (PAF), and demonstrated that 68 (28%) patients had AF initiated by ectopic beats (73 foci, 20%) from non-PV areas, including the LA posterior free wall (28, 38.3%), superior vena cava (27, 37.0%), crista terminalis (10, 13.7%), LOM (6, 8.2%), CS ostium (1, 1.4%), and interatrial septum (1, 1.4%). Liu CM *et al*. [25], investigated 254 patients with non-PAF, demonstrating that AF was induced in 102 foci in 67 (26%) patients, including the LA posterior free wall or left atrial appendage (LAA) (20, 19.6%), superior vena cava (23, 22.5%), right atrium or crista terminalis (12, 11.8%), LOM (20, 19.6%), CS (16, 15.7%), and interatrial septum (11, 10.8%). AF is documented to originate more frequently from the distal segment than the proximal segment of the LOM. Conversely, ectopic AT tends to arise more frequently from the proximal segment of the LOM, particularly in locations near the CS [7].

## 3. Role of the LOM as a Substrate for AF 

The LOM may be important not only as a focus for AF, but also as a substrate for AF. Dave AS *et al*. [21], studied 61 patients with recurrent AF or flutter after PV isolation and demonstrated left PV reconnection may occur via epicardial connections through the VOM, albeit in a minority of cases [26]. More recently, Barrio-Lopez MT, *et al*. [27] examined 534 consecutive patients with AF undergoing radiofrequency (RF) ablation and demonstrated that 81 epicardial connections were observed in 72 (13.5%) patients and half were connections between the left PVs and the LOM. Comparatively, patients with epicardial connections exhibited lower acute success in pulmonary vein isolation than those without epicardial connections (99.1% versus 86.1%; *p* < 0.001). After adjustment for age, sex, and various other factors, patients with epicardial connections demonstrated a higher risk of atrial tachyarrhythmia recurrence compared to those without (hazard ratio, 1.7 [95% CI, 1.1–2.9]; *p* = 0.04) [27]. Persistent left superior vena cava (PLSVC) is observed when the left anterior cardinal vein does not recess to the LOM and remains patent [28, 29]. PLSVC exhibits a relatively low prevalence in the general population and is infrequent among patients referred for RF catheter ablation of AF. However, it still may be the origin of AF [30, 31], and PLSVC isolation during catheter ablation is associated with a reduction in AF recurrence [32, 33].

Makino M *et al*. [14], examined 28 postmortem human hearts including 3 cases with Paroxysmal AF (PAF) and the other 3 with chronic AF (CAF) to demonstrate the various connections between the LA and the LOM. The LOM was observed in 25 hearts (89%). Close connections near the CS juncture were observed in 18 cases (64%) (Non-AF, 12 cases [63%]; PAF, 3 cases [100%]; CAF, 3 cases [100%]). Distant connections were noted in 16 cases (72%) (Non-AF, 12 cases [63%]; PAF, 3 cases [100%]; CAF, 1 case [33%]). Furthermore, left PV to LA junctional connections were seen in 18 cases (72%) (Non-AF, 13 cases [68%]; PAF, 3 cases [100%]; CAF, 2 cases [67%]). Additionally, apart from these three connections, multiple and wide connections extending continuously were observed in 9 cases (36%) (Non-AF, 6 cases [31%]; PAF, 3 cases [100%]; CAF, 0 cases [0%]). This anatomical diversity has also been electrophysiologically demonstrated by Han S *et al*. [20]. In their investigation, a single connection between the Marshall bundle (MB) and CS muscle sleeves was observed in 11 out of 64 patients (17.2%). Recordings from the MB revealed distinct potentials with a proximal-to-distal activation pattern during sinus rhythm. Double connections to both the CS and LA around the left PVs were observed in 23 out of 64 patients (35.9%). Post ablation of the distal connection, MB recordings displayed typical double potentials as a single connection. Additionally, 30 out of 64 patients (46.9%) exhibited multiple connections, with the earliest activation occurring in the middle of the MB during sinus rhythm. Activation patterns demonstrated variability and irregularity in each patient. During AF, rapid and fractionated complex activations were consistently observed in this group. These anatomical and electrophysiological findings suggest the potential formation of complex multiple myocardial connections between the LOM and LA, potentially contributing to the development and maintenance of reentrant circuits underlying AF [7, 8, 9].

In sustained AF induced by chronic pacing, a significant gradient of activation rate was demonstrated in 6 dogs by Wu TJ *et al*. [10]. The mean cycle length was shorter in the LOM (84 ± 5 ms), the left inferior pulmonary vein (LIPV) (81 ± 4 ms), and the left superior pulmonary vein (LSPV) (85 ± 7 ms) than the LA free wall (96 ± 5 ms) and the RA fee wall (126 ± 17 ms). The dominant frequency was highest in the LOM and the PVs (range 11.2 to 13.3 Hz), followed by the LA (*p* = 0.001) [10]. Similar findings are also observed in the human heart. Kamanu S *et al*. [34], recorded activations in the LOM in 4 persistent AF and 2 permanent AF cases, demonstrating that the activation at the VOM (CL = 140 ± 31 msec) is significantly shorter than that at other sites in the atrium (*p* < 0.05), and the dominant frequency at the VOM (9.71 Hz ± 1.52 Hz) is also significantly higher than that at other sites in the atrium (*p* < 0.0001) during AF. These data suggest that the LOM region may lead AF in some cases [34].

## 4. Role of the LOM as a Substrate for AT

The LOM may more commonly serve as part of a circuit for reentrant ATs after AF ablation. We have described two types of MB-related reentrant ATs [11]: MB-related perimitral flutter and MB-related localized AT. Out of 199 scar-related ATs in 140 patients undergoing at least one prior procedure (mean: 2.9 ± 1.5) including pulmonary vein isolation (PVI), either as a standalone procedure or combined with mapping and ablation of AT, 60 (30.2%) ATs were diagnosed as LOM-related ATs including 31 (15.6%) perimitral flutters and 29 (14.6%) localized reentries. Hayashi and colleagues [12] reported that following PVI or valve surgery, LOM-related perimitral flutter accounted for up to 11% of perimitral flutter cases. After multiple procedures, residual LOM-related epicardial connections are more likely to be included in the AT circuit. We followed 147 patients with AF-ablation related ATs mapped by Rhythmia™, and examined 44 patients who received redo procedures, and the AT-maps during the redo-procedure were compared to the original AT circuit to investigate the mechanism of AT-recurrence. When the original AT is focal AT, cavo-tricuspid isthmus (CTI)-dependent AT, or non-anatomical macroreentrant AT, the circuit of the recurrent AT is usually different from the original AT-circuit. However, when the original AT is perimitral flutter or roof-dependent macroreentrant AT, the circuit of the recurrent AT is similar to the original AT in 57.7% and 44.4%, respectively [13]. In addition, epicardial structures are included in the circuit in 51.2% of recurrent ATs. Especially in perimitral flutter, the CS/VOM-system is involved in the circuit in 75% of redo cases13. Similar to our findings, several studies have demonstrated the difficulty in achieving transmural linear lesions across the mitral isthmus (MI) and the LOM [35, 36, 37]. Anatomical and histological findings [3, 14] have demonstrated that the LOM has multiple connections with the LA and is insulated from its surroundings by fatty tissue. These structures may also act as substrate for ATs.

**Fig. 4.**
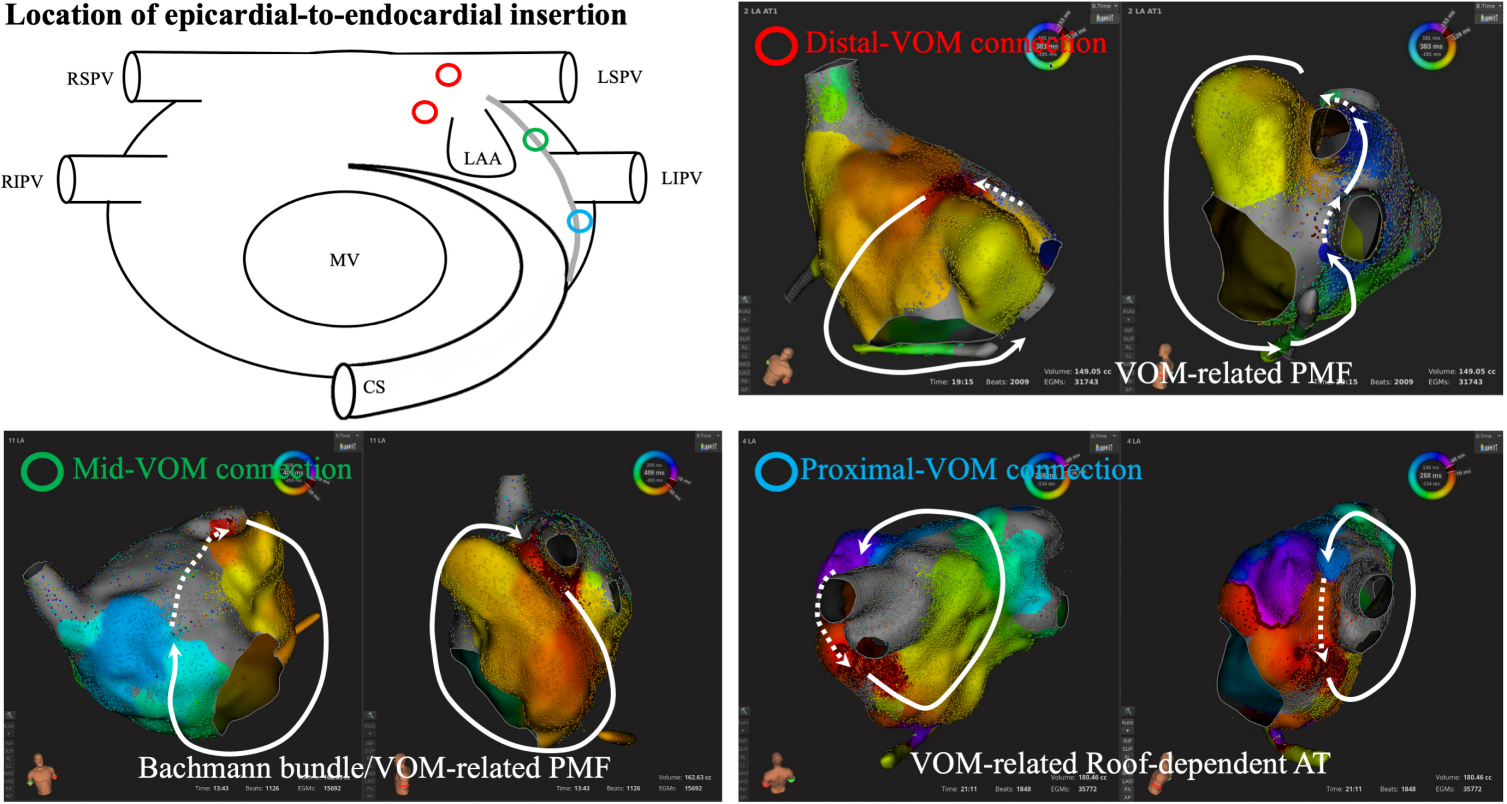
**Location of epicardial-to-endocardial insertion from the ligament of Marshall**. Diverse connections between the ligament of Marshall and endocardial left atrium are observed along the ridge between the LAA and LPV. Centrifugal activation is identified at the endocardial insertion point of the epicardial structures, which is one of the clues to predict LOM-related ATs. AT, atrial tachycardia; CS, coronary sinus; LAA, left atrial appendage; LIPV, left inferior pulmonary vein; LSPV, left superior pulmonary vein; MV, mitral valve; PMF, perimetral flutter; RIPV, right inferior pulmonary vein; RSPV, right superior pulmonary vein; VOM, vein of Marshall; LPV, left pulmonary vein; LOM, ligament of Marshall.

LOM-related ATs should be strongly suspected when arrhythmia recurs after extensive ablation including endocardial mitral isthmus ablation [13]. When 3D-mapping systems display a centrifugal activation from the ostium of the LAA and the top of the ridge [38], mid-low regions of the ridge [39], or the neighboring areas around the LIPV, reentrant (pseudo-focal) ATs using the LOM [40, 41] should be differentiated from true focal ATs (Fig. 4).

## 5. Radiofrequency Ablation to the LOM

RF-ablation of the LOM can be performed while mapping this structure either epicardially or endocardially. Hwang C *et al*. [4] described an endocardial ablation technique involving cannulation of the VOM with a mapping catheter for anatomical guidance during ablation and confirmation of its success. A 1.5–2.7 Fr mapping catheter can be placed in the VOM through the CS to record LOM-electrograms. In approximately half of the patients, double potentials were recorded, indicating LA activation as initial potentials and LOM potentials as secondary. By targeting these potentials, RF lesions may be created endocardially at the left lateral ridge, corresponding to the epicardial LOM. The area closest to the LA endocardium and the LOM is situated in the inferior region of the LA, just below the LIPV ostium. Endocardial RF applications in this region can eliminate LOM potentials.

One the other hand, VOM cannulation is not always successfully performed because of various anatomic and technical reasons. When the VOM is not visible or endocardially not accessible, an epicardial approach following Sosa’s technique [42] may be an alternative strategy for successful mapping and ablation of this structure. Berruezo A *et al*. [43], demonstrated the effects of transthoracic epicardial ablation of the LOM in four patients experiencing a recurrence of highly symptomatic drug-refractory peri-mitral flutter resistant to endocardial and CS mitral isthmus ablation. However, this approach is invasive and may pose challenges for non-experienced institutions.

## 6. (i) EI-VOM: Impact on MI Ablation

Attaining complete MI block using RF poses a challenge, with success rates reported between 31% to 92% [44, 45, 46, 47, 48, 49], often necessitating RF applications within the CS (59–91%). The complexity of achieving complete MI block may be linked to several anatomical limitations, including a thicker and longer MI [50, 51], and anatomical variations between the MI and epicardial structures, such as the CS/VOM and circumflex artery [52, 53, 54]. Piorkowski C *et al*. [55], conducted simultaneous epicardial and endocardial mapping in 55 patients who had undergone at least two prior attempts of endocardial catheter ablation and continued to experience symptomatic paroxysmal AF, persistent AF, or AT despite prior PVI. The study demonstrated the completion or addition of a total of 165 linear ablation lines during the procedure, with 63 out of 165 lines (38%) requiring epicardial ablation to achieve continuity. Notably, all 9 classical MI lines drawn during the procedure required epicardial ablation.

In addition, the assessment of the achievement of MI block is challenging. It has been reported that pseudo-MI block, in which a conventional pacing maneuver displays complete MI block, but high-resolution mapping or a catheter placed in the VOM demonstrates incomplete MI block, is observed in approximately 20–30% of cases [35, 36, 56]. The main mechanism of this mis-diagnosis is residual epicardial conduction with endocardial block [13]. One of the technical approaches to overcome the difficulty in achieving complete MI-block is to select different linear lines such as an anterior line or anteroseptal line [57, 58, 59, 60]. However, these lines are strongly associated with the incidence of bi-atrial tachycardias [61, 62, 63, 64], which are usually refractory to ablation therapy. If complete endocardial-to-epicardial block can be achieved, an ideal line theoretically exists in the posterior MI region connecting the lateral mitral annulus with the left PVs. This area is typically the latest activation site of the LA during sinus rhythm (SR), and establishing bidirectional block in this specific region will not disrupt physiologically normal conduction during SR. Additionally, this line can avoid the risk of biatrial atrial tachycardias. However, the augmentation of RF power or contact force to create transmural and durable lesions at this site from the endocardial side may simultaneously elevate the risk of complications, such as cardiac effusion or tamponade.

An epicardial approach such as EI-VOM may be an optional strategy to add to RF to eliminate conduction through this epicardial structure [13, 65, 66, 67, 68, 69]. This combined approach, using EI-VOM along with RF, has been reported to achieve an exceptionally high success rate of 98–100% for the achievement of mitral isthmus block [65, 66, 70, 71]. We have first demonstrated this in patients with perimitral flutter (PMF). Our study involved 103 consecutive patients with PMF who underwent high-resolution mapping, including an initial group of 71 patients treated solely with RF ablation (RF-group), followed by a subsequent group of 32 individuals who underwent EI-VOM followed by RF applications on both the endocardial and epicardial mitral isthmus (EI-VOM/RF-group). Although PMF similarly terminated in both two groups, the tachycardia terminated with EI-VOM alone in 68.6% of cases in the EI-VOM/RF-group. The median duration of RF for termination of conversion of AT was notably shorter [0 (0–6) s] in the EI-VOM/RF-group compared to [312 (55–610) s] in the RF-group (*p* < 0.0001). Similarly, the duration for achieving complete block of the mitral isthmus was shorter in the EI-VOM/RF-group [246 (0–663) s] than in the RF-group [900 (525–1310) s, *p* < 0.0001]. During the one-year follow-up, the EI-VOM/RF-group exhibited fewer recurrences [6/32 (18.8%)] compared to the RF-group [29/71 (40.8%), *p* = 0.04]. Multivariate analysis revealed that only EI-VOM was significantly associated with a lower risk of AT recurrence (hazard ratio = 0.35, *p *= 0.018). Nakashima T *et al*. [66], conducted a comparative analysis between two groups of patients undergoing a first attempt at posterior myocardial infarction (MI) ablation: those receiving adjunctive EI-VOM (n = 152) versus those treated with RF alone (n = 110). The study compared the rates of acute MI block and observed MI reconnection during subsequent procedures between these two groups [66]. The EI-VOM group exhibited a higher frequency of achieving acute MI block (98.7% [150/152]) compared to the RF alone group (63.6% [70/110]; *p <* 0.001). Moreover, the EI-VOM group required a shorter duration of RF application (5.00 [3.00–7.00] minutes) in contrast to the RF alone group (19.0 [13.6–22.0] minutes; *p <* 0.001). Among the 220 patients who achieved MI block during the initial procedure, 81 underwent a repeat procedure during follow-up. A lower percentage of patients in the EI-VOM group required repeat procedures compared to the RF alone group (EI-VOM group: 23.3% [35/150] vs. RF alone group: 65.7% [46/70], respectively; *p <* 0.001). Notably, a significantly larger proportion of patients demonstrated sustained or durable MI block in the EI-VOM group (62.9% [22/35]) compared to the RF alone group (32.6% [15/46], respectively; *p *= 0.008). Laredo M *et al*. [72], focused on the durability of MI block over time in 24 consecutive patients who underwent EI-VOM for persistent AF or PMF (index procedure) and repeat catheter ablation for recurrent AT or AF. Among the 20 patients who initially achieved complete MI block at the index procedure, reconnection of the MI was observed in 9 patients (25%). The median low bipolar voltage (<0.05 mV) area in the VOM region (13.1 ${\mathrm{cm}}^{2}$ [8.1–15.9] ${\mathrm{cm}}^{2}$) was comparable to the area observed immediately after EI-VOM (median 12.4 ${\mathrm{cm}}^{2}$ [7.6–15.7] ${\mathrm{cm}}^{2}$). A recent meta-analysis including 1322 patients in 10 studies, demonstrated that EI-VOM combined with RF-catheter ablation significantly increased the rate of bidirectional mitral isthmus block compared with RF-catheter ablation alone in AF patients [73].

While adopting EI-VOM as a first-line therapy for all PMF may still be controversial, this approach should be considered before alternative epicardial approaches [43, 74] in cases of refractory PMF due to its less invasive nature and fewer complications.

## 6. (ii) EI-VOM: Impact on AF Ablation

While most publications detail the methodology of EI-VOM [74], or represent single arm data [26] without a comparator, one recent meta-analysis provides compelling evidence that adjuvant EI-VOM reduces the recurrence rate of AF and/or AT in patients with persistent AF. Liu CM *et al*. [25] have reported the long-term efficacy of EI-VOM as an adjunctive treatment to the conventional ablation strategy for non-paroxysmal AF and concluded that EI-VOM decreased the risk of AF recurrence by 80%. Recently, a prospective, multicenter, randomized, controlled trial, the VENUS-AF trial has been published, where the superiority of concomitant EI-VOM to conventional catheter ablation alone in reducing AT/AF recurrence and burden was demonstrated [69]. The study included 343 patients with symptomatic persistent AF comparing a group with catheter ablation alone (n = 158) vs. that with EI-VOM and catheter ablation (n = 185). Although 30 (16.2%) patients in the EI-VOM plus catheter ablation group did not receive successful EI-VOM, fewer recurrences were observed after a single procedure and off antiarrhythmic drugs in the EI-VOM plus catheter ablation group (49.2%) than the catheter ablation alone group (38%). Importantly, sub-analysis of the VENUS trial demonstrated that the outcome of the procedure was significantly better in patients when mitral isthmus block was achieved [75]. Derval N *et al*. [76], have also reported the clinical impact of EI-VOM in a prospective, single-center study. This approach, termed the Marshall-PLAN, involved the elimination of the Marshall bundle through EI-VOM, along with PVI and comprehensive anatomical ablation including linear lesions across the mitral isthmus, roof, and CTI. A total of seventy-five consecutive patients diagnosed with persistent AF were enrolled in the study. EI-VOM was successfully completed in 69 patients (92%), and the full Marshall-PLAN lesion set was accomplished in 68 patients (91%). After 12 months, 54 out of 75 patients (72%) remained free from AF/AT following a single procedure without the use of antiarrhythmic drugs in the entire cohort. Within the subset of patients who received the complete Marshall-PLAN lesion set (n = 68), the success rate after a single procedure was 79%. Presently, they conducted a prospective, randomized, multicenter study comparing the Marshall-PLAN strategy against PVI for persistent AF. Although the follow-up is still ongoing, the interim report presented in the late-breaking session in EHRA2023 indicates the superior efficacy of the Marshall-PLAN strategy. The trial included 120 patients with symptomatic persistent atrial fibrillation for more than one month. The total radiofrequency time was significantly longer in the PVI group (29 minutes) compared to the Marshall-Plan group (23 minutes; *p* < 0.001). The full lesion set was successfully achieved in 88% of patients with the Marshall-Plan strategy and 98% with PVI only. In an intention-to-treat analysis, the recurrence of arrhythmias after an average follow-up of 10 months was significantly higher in the PVI group than in the Marshall-Plan group (18 vs. 8 patients; *p* = 0.026). In addition, Lai Y *et al*. [77], compared a ‘2C3L’ approach including bilateral PVIs and 3 anatomical lines on the mitral isthmus, roof and CTI, performed solely by RF-ablation with the same approach but with EI-VOM and RF-ablation (upgraded ‘2C3L’ approach) for the treatment of persistent AF [77], demonstrating that a better outcome at 12-month follow-up was observed in patients treated with the upgraded ‘2C3L’. A randomized clinical trial ‘PROMPT-AF’ (Prospective randomized comparison between upgraded ‘2C3L’ vs. PVI approach for catheter ablation of persistent AF) is now also underway [78].

While recent reports have demonstrated a preference for adjunctive EI-VOM over conventional RF alone [69, 76, 77], a conflicting result was initially reported in the MARS trial, a prospective multicenter randomized controlled study. In this trial, 80 patients with persistent AF undergoing repeat catheter ablation were randomly assigned to either catheter ablation alone or catheter ablation combined with EI-VOM. Although EI-VOM was successfully performed on 90% of the patients, there was no significant difference in the incidence of achieving MI-block or freedom from AF/atrial tachycardia 12 months after the procedure between the two groups [79]. The impact of EI-VOM on clinical outcomes may depend on the study population and the additional ablation strategies used in conjunction with EI-VOM. Therefore, a meticulous examination of long-term outcomes is warranted.

## 7. Main Approaches for EI-VOM

**Fig. 5.**
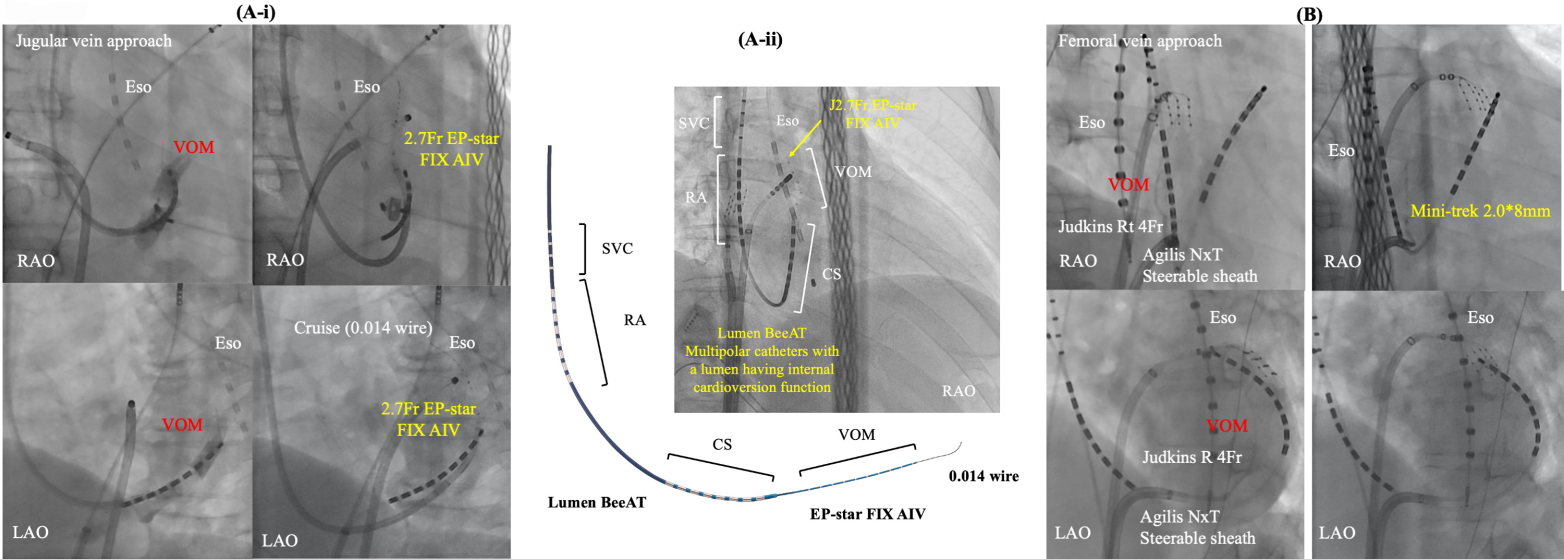
**Two main approaches for EI-VOM**. Jugular vein approach with a guide sheath (A-i) or without (A-ii) and femoral vein approach (B). CS, coronary sinus; Eso, esophageal temperature monitoring; EI-VOM, ethanol infusion to the vein of Marshall; LAO, left anterior oblique view; RA, right atrium; RAO, right anterior oblique view; SVC, superior vena cava; VOM, vein of Marshall.

With a grasp of fluoroscopic anatomy and utilizing angioplasty tools, the VOM ethanol technique is not complex and achieves success in approximately 90% of cases [71, 80, 81, 82, 83]. Failure should be limited to patients without a VOM. Two main approaches have been reported as shown in Fig. 5. Valderrábano M *et al*. [74, 84], first delineated the safety and efficacy of ethanol infusion in the VOM during catheter ablation in 14 patients with AF undergoing PVI [74]. Successful cannulation of the VOM was achieved in 10 out of 14 patients (71.4%) with a reduction in ablation time for PVI, without encountering any complications. Initially, CS cannulation was accomplished by introducing a 7 Fr sheath via the right internal jugular vein (Rapido CS-EH, Boston Scientific, St. Paul, MN, USA). A subsequent CS venogram was conducted to visualize the ostium of the VOM, using a balloon to obstruct the CS ostium.

Efforts were made for sub-selective cannulation of the VOM using a left internal mammary artery (LIMA) or Judkins-right (JR) angiographic guide catheter ﻿(BMW, Abbott, Abbott Park, Illinois, USA), or a sub-selective catheter for CS branch cannulation (Rapido IC-90, Boston Scientific). Radiographic contrast was injected to confirm engagement in the VOM while Marshall electrograms were collected using a slim catheter (1.5 Fr–2.7 Fr). Once engagement was established, an angioplasty wire was inserted into the VOM, followed by the advancement and inflation of a pre-loaded angioplasty balloon (8 mm length, 2 mm nominal diameter) in the VOM. A selective VOM venogram was obtained by injecting contrast into the balloon lumen, revealing the VOM distribution. As described in the original report, two separate 1 mL injections of 100% ethanol were administered, each over a 2-minute interval. Subsequent studies have adopted and replicated this procedural approach [26, 68]. Recently, a multipolar catheter featuring an internal cardioversion function has been introduced (Lumen BeeAT, Japan Life Line, Tokyo, Japan). This catheter is advanced into the CS with guidance from a 0.035-inch guidewire. Subsequently, the vein of Marshall (VOM) is accessed by the 2.7 Fr catheter (EP-star FIX AIV, Japan Life Line, Tokyo, Japan) with a guidance of a 0.014-inch wire (﻿Whisper 0.014, Abbott or Sion Blue 0.014; Asahi). Finally, the catheter covers the superior vena cava, RA, CS, and VOM simultaneously, also facilitating efficient exploration of non-pulmonary vein foci after internal cardioversion.

On the other hand, a femoral approach is originally described by Hwang C *et al*. [4]. We have also modified this approach [85]. In this method, a steerable long sheath Agilis NXT (Abbott Inc, St. Paul, MN, USA) is inserted into the CS from the right femoral vein, guided by an ablation catheter. Subsequently, a selective venogram of the VOM is performed using a 5 Fr angiography catheter within the CS via the steerable sheath. At each location within the CS, a small amount of contrast is injected through the LIMA catheter or JR catheter to locate and engage the ostium of the VOM. In cases where the VOM is not identified, a balloon occlusion venogram of the CS is performed to facilitate VOM identification. VOM-cannulation was successful in 50/54 (92.5%) pts in this study. However, VOM was not visible in one patient, and guidewire insertion was not possible due to small VOM. Select an appropriately sized balloon (1.5–2.5 mm diameter and 6–15 mm length, 145 cm MINI TREK; Abbott, USA) based on the VOM size to occlude its ostium. After confirming balloon occlusion and VOM distribution through contrast injection, slowly inject 0.5–3 mL of ethanol (96% ethanol 10 mL, 8.08 gr, 808 mg/mL) over 1 minute, followed by a repeat of selective venography of the VOM. We empirically administer a total of 6 to 12 mL of ethanol. Kamakura T *et al*. [83], reported that out of a total of 713 patients who received EI-VOM with the femoral approach, the procedure was failed in 79 patients (11.1%) due to the following reasons: (1) nonidentification of the VOM in 44 patients (6.2%); (2) non-cannulation of the VOM in 11 patients (1.5%); (3) ethanol infusion inside a wrong vein (inferior LA vein: 0, LAA vein: 10, undefined vein: 2) in 12 patients (1.7%); (4) CS dissection in 6 patients (0.8%); (5) persistent left superior vena cava in 4 patients (0.6%).

## 8. Various Distribution of the VOM

**Fig. 6.**
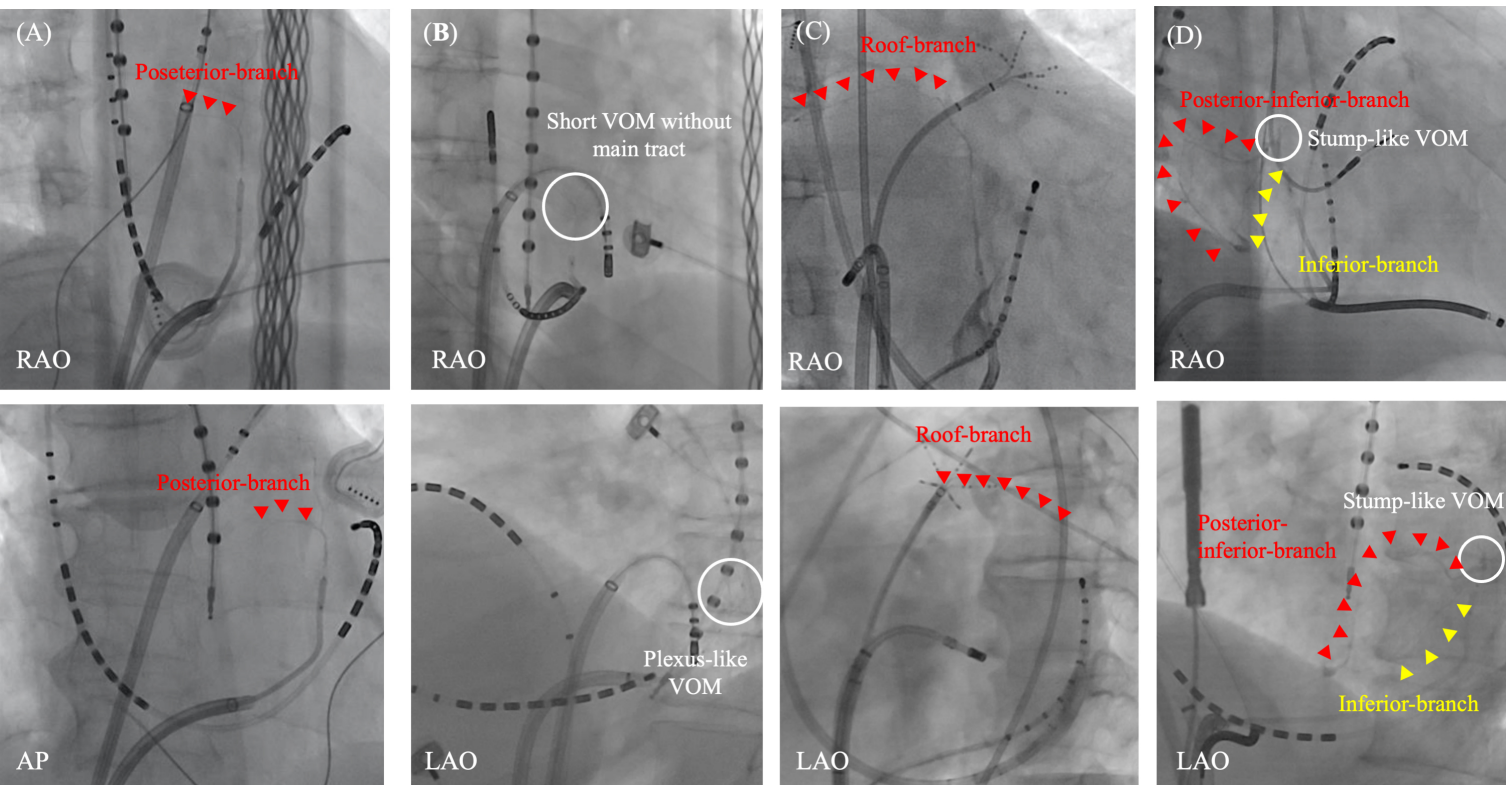
**Various distribution of VOM branches**. Various distribution of VOM branches are observed. A posterior branch from the main VOM-tract (A). A plexus-like short-VOM without a main tract (B). A roof branch from the distal part of the main VOM-tract (C). Stump-like VOM with two branches from the VOM-ostium; one is a posterior-inferior branch and the other is inferior branch (D). AP, antero-posterior view; LAO, left anterior oblique view; RAO, right anterior oblique view; VOM, vein of Marshall.

Venous distribution is varied and abundant collaterals are observed in LA. However, a constant pattern may be recognized and summarized in the report by Valderrábano M *et al*. [16]. Beginning with the CS ostium, the LA veins include: (1) a septal vein; (2) a second, inferior atrial vein; (3) the VOM; (4) LAA veins; and (5) an anterior roof vein. Additionally, LA veins not connected to the CS are observed, including (6) roof veins and posterior wall veins commonly linked with (7) extracardiac collaterals. While the VOM is consistently the most frequently found LA vein, identified as the vein arising at the level of the valve of Vieussens (ostial to it), various venous distributions and collaterals are still observed (Fig. 6). In our study, the distance from the CS ostium to the VOM ostium was 4.25 ± 2.57 cm, displaying substantial variability. The VOM was commonly identified as a true atrial vein, with variable branching into small venules observed in most patients (78.2%). Length of the VOM before branching was 2.99 ± 1.82 cm. However, in certain cases, a venous plexus (10.4%) at the VOM origin or a stump (12.2%) with few or no atrial branches and an abrupt transition from the CS origin into a cul-de-sac were noted. The VOM was observable up to the LIPV in the majority of patients (72.8%), extending further to the LSPV in 9.6% of cases. However, in some instances, the VOM was smaller, not even reaching the LIPV (17.6%). Communication between the VOM and left PVs, as demonstrated by the presence of contrast drainage during VOM-venography, was observed in 37.7% of cases. It is essential to visualize the complete distribution of the VOM, including distal branches and collaterals, before injecting ethanol. The impact of EI-VOM is associated with this distribution. Kamakura T *et al*. [86], characterized the distribution of low voltage observed in the LA after EI-VOM in 114 AF patients. Although the two most frequently impacted segments were the inferior segment of the ridge (82.5%) and the first half of the mitral isthmus (pulmonary vein side) (92.1%), these areas were not affected when the VOM had no branches. A residual gap on the mitral isthmus was more frequently observed at the annular side [66]. Interestingly, an effect of EI-VOM was observed on the lower left posterior wall in 19.3% adjacent to the esophagus. EI-VOM may be an optional strategy for safely and effectively eliminating arrhythmogenic epicardial structures around the posterior LA region adjacent to the esophagus in cases where RF applications are challenging due to frequent temperature rise [87]. Although low voltage extending to the lower dome, posterior wall, or the entire LA can be anticipated by a visible VOM anastomosis with roof or posterior veins, this does not always guarantee a significant impact in these areas [86].

Assessing the VOM anatomy before the procedure may help reduce radiation duration, contrast medium volume, and the risk of complications. While visualizing the VOM by computed tomography (CT) imaging is generally challenging, an optimized CT acquisition protocol dedicated to VOM visualization has been reported by Takagi T *et al*. [88]. In this protocol, a contrast bolus of 50 mL of 100% iodine contrast media was injected at 5 mL/s, followed by 40 mL of 100% iodine contrast at 3 mL/s, and then by 20 mL of 100% saline at the same rate. The acquisition was set at a fixed 20-second delay after detecting the enhancement of LA chamber (100 Hounsfield Units [HU] threshold). Administration of sublingual nitroglycerin before CT acquisition was required to maximize coronary venous flow [88, 89]. The VOM distribution was more frequently detected with this dedicated protocol than with a conventional protocol (63% vs. 35%). The VOM emerged in the superior portion of the cross-sectioned CS in 68% and in the postero-superior portion in 32%.

## 9. Complications of EI-VOM

Kamakura T *et al*. [83], reported successful EI-VOM in 634 out of 713 patients scheduled for the procedure (88.9%). VOM perforation, characterized by iodine extravasation into the pericardial space, was observed in 20 cases (2.8%), along with pericarditis in 13 instances (1.8%), defined by chest pain and limited pericardial effusion. A total of 14 serious complications (2.0%) were documented: 7 (1.0%) cases of tamponade, 6 of which were delayed and necessitated pericardiocentesis performed several days post-procedure (range: 7–106 days), 4 (0.6%) strokes, 1 (0.1%) incident of anaphylactic shock, 1 (0.1%) atrioventricular block, and 1 (0.1%) left appendage isolation. Only 4 of these severe complications occurred during the procedure. The incidence of cardiac tamponade was notably higher in patients with VOM perforation compared to those without (10% vs. 0.7%, *p* = 0.014), with serous effusion in 4 out of 7 cases (57%). While the overall complication rate remains low, delayed cardiac tamponade emerges as a distinctive complication following EI-VOM. Another study involving 129 patients undergoing EI-VOM also indicated a relatively high incidence of delayed cardiac tamponade at 3.1% [90]. Generally, the occurrence of delayed cardiac tamponade following RF catheter ablation of AF is relatively infrequent. Out of 27,921 procedures conducted in 21,478 patients, 45 cases (0.16%) of delayed cardiac tamponade were observed. Patients developed delayed cardiac tamponade at a median of 12 days (range: 0.2 to 45 days) following the ablation procedures [91]. Hemorrhagic pericardial fluid was observed in most of them (n = 36, 80%). Although both sealed micro perforations in the atrium and subacute pericarditis, like Dressler’s syndrome, may be associated with the mechanism of delayed cardiac tamponade, the latter factor appears to be more commonly linked to cardiac tamponade after EI-VOM, based on the observations of a higher incidence of pericarditis and delayed cardiac tamponade, and more serous rather than hemorrhagic pericardial effusion, in contrast to acute cardiac tamponade.

In approximately 30% of cases where EI-VOM is performed, localized staining may be observed. This results from the leakage of contrast medium from ruptured venules, likely caused by dissection, balloon inflation, guidewire manipulation, or high-pressure ethanol infusion. However, no significant difference is reported to be observed between cases with localized staining and those without in the incidence of complications, the achievement and durability of MI-block, the area of low-voltage area, and the clinical outcome [70].

## 10. Conclusions

The LOM is an epicardial structure composed of sympathetic nerves, veins, and multiple muscular bundles connecting to the LA, and insulated from its surroundings by fatty tissue. The LOM is responsible for both focal and reentrant arrhythmias in patients with AF, and serves as a trigger and/or driver of AF. Further, the LOM is frequently included in the circuit of complex ATs, especially in cases where endocardial ablation has been performed at the mitral isthmus. EI-VOM is an efficient and safe approach to aid elimination of this arrhythmogenic structure.
